# An Enemy in the Camps

**Published:** 1863-09

**Authors:** 


### AN ENEMY IN THE CAMPS.

A large portion of the national army is in a section of country notorious for the production of malaria, an enemy more insidious and more dangerous than all the physical forces that the rebels can bring to bear against the loyal troops. The records of the human race are filled with instances which prove the truth of which we speak. The rapid and overwhelming disaster to the forces of Sennacharib is familiar to readers of history, but it was scarcely more rapid or more crushing than the malarious destruction of the French army in 1528, which, while preparing to besiege the forts protecting Baiæ, was almost totally destroyed by disease. Of 28,000, but 4,000 remained alive, and they were helpless.

Until within a comparatively recent period the mortality in the British navy was 1 in every 8, then it became 1 in 13. The ship Columbus, with a crew of 400, lost in one season on a South American station 200 of her men. The causes of this state of things were obvious to all hygienic authorities, and they devised means of prevention that have reduced the mortality to 1 in 72, or to a ratio equal to that of the healthiest of the rural districts of England. Of these means we shall presently speak. We call attention now to facts connected with military matters. In Wellington's Peninsular campaigns from January 1811 to 1814, the battles of Albuera, one of the most desperate and bloody of the Peninsular War, Salamanca, Vittoria, the Pyrenees, Nivelle, Nive, Orthes, and Toulouse were fought, and Badajos, Ciudad Rodrigo and San Sebastian were stormed, besides many skirmishes not included in the list of battles, the entire loss in battles was 2,550, while that from sickness was 7,257. In the war with Burmah the loss by military forces was thirty-five per thousand, and from sickness the loss was four hundred and fifty per thousand. In the Crimean war, while the armies were encamped in the valley of Varna, in the midst of "large shallow lakes, surrounded by level spongy lands, indented with little hollows, dried and cracked by the recession and evaporation of the winter floods—low brushwood, rank in vegetation, bounding uplands, a deficiency of potable water, with a high temperature," we have all the elements of a devastating sickness. In the high temperature of the day, heavy masses of steam spread themselves over the camps, and at night heavy, chilling dews invaded every part of the camp, and carried the poison to every sleeper. The "tents were thin and permeable, the rations execrable," and no protecting care was exercised. A medical philosopher with these facts before him knows the result already. The French and Turks suffered terribly. Macleod says the hospitals recalled the horrors of Boccaccio. Half of the army of Espinasse, in the Drobrutcha, disappeared as by a whirlwind, and the panic among the survivors was beyond description. Cholera, intermittent and remittent fever, typhus and dysentery took possession of the camps—the encampment was broken up, and the army fled precipitately from the scene of the disaster—but the enemy retained possession of the men, and the horrors of Varna continued to follow them. The survivors continued for years to feel the dreadful visitation of the Drobrutcha, and those who seemed to have passed unscathed showed in subsequent wounds that the seeds of the poison of the camp had been merely latent. Among the English, there were in three months 897 deaths from cholera, and 75 from dysentery and diarrhœa. Dr. Aitkin says: "My estimates lead with still greater force to the conclusion, that the amount of sickness at Varna was greater than that of the French army in Spain, and nearly as great as the army of Portugal while engaged in very active campaigns, and this, too, though not a soldier in Lord Raglan's army had fired a shot." From October, 1854, to April, 1855, the army of 23,775 men, lost 9,248 by sickness, and 608 by wounds. In the last six months of the Crimean campaign, including the final assaults which carried Sebastopol, the French had 21,957 men wounded, and 101,128 cases of sickness.

Now, the great mass of this sickness was avoidable, and should have been prevented. In the Italian campaigns, Napoleon guarded his troops against the disasters connected with localities. He never encamped his armies in the neighborhood of malarial sources when he could avoid it, and when compelled to make such an encampment, he always built fires at night between his army and the sources of this poison. We earnestly wish that we could engrave in vivid letters upon the memories of those who have the management of the American camps the following truths of Sir Baltingall. He says: "The experience of all ages has proved that the neighborhood of marshes, grounds subject to overflow by large rivers, surrounded by foul stagnating water, or low places covered with wood, are most injurious to health, and the noxious effluvia arising from these situations are augmented in proportion to the heat of the climate or the season of the year."

In all perils of this kind, the camp should be pitched so that the evening wind will blow the marsh air in an opposite direction from the camp. When this cannot be done, fires should be burned all night between the sources of the malaria and the camp. Malaria never acts in daytime nor at night upon a wakeful person in motion. Sentinels may walk in safety, where a sleeping army may be almost destroyed. No garbage should be permitted about camps. Let it be buried or thrown into running water. It should not be burned in camp.

As the sun climbs the ecliptic, he scatters the seeds of sickness northward. Rio Janeiro, Pernambuco, Cuba, Tampico, Vera Cruz, New Orleans, Vicksburg, and places still further northward take their turn. The cause of pestilence is now incubating in the regions held by our armies. They can and must be protected from the pestilence that walks in darkness only to those who wilfully shut their eyes. The men must be well fed, for there is a great truth in the aphorism, 'that the first condition of health is the good condition of the stomach.' M. Worms, in his work, "*Des Maladies de la Province de Constantine*,' says: 'Those who are well nourished pass through or even sojourn with impunity in localities where others meet with disease and death. In the army, where soldiers and officers are exposed to the same morbid influences, the average deaths are one in twelve of the former to one in fifty-four of the latter. The officers, by the proper nourishment and the use of fermented liquors, sustain the vital energy, which has a tendency to fall into inertia, and so escape the effects of malaria, which makes ravages around them. The Commissioners of Inquiry of the British army at Sierra Leone found that the main cause of the fearful mortality from diseases of the digestive organs there, two-fifths of the cases having proved fatal, arose from the use of salt rations, and that by the substitution of a fresh meat diet, the mortality from these diseases was reduced to one-tenth of its former amount. Haliday's testimony is to the same effect."

The British Government has great faith in the preventive effects of Quinine, and the laws require its use as a prophylactic in all exposed situations in malarious regions. It is to the use of this article, in this way, that the improvement in the health of the British navy, on duty on tropical coasts is due.

We have indicated the perils of our armies in the South and the means of salvation. We appeal to the officers to see to the protection of the brave men committed to their charge.—*Sanitary Reporter.*

